# Supplementary material for: Are carbon emissions trading and green financial instruments synergistic? -Comprehensive quantitative research based on content analysis
Source: PLoS One. 2024 Mar 7;19(3):e0298601. doi: 10.1371/journal.pone.0298601 (PMC10919601; doi:10.1371/journal.pone.0298601)
Supplement: S1 Data — (ZIP) [file pone.0298601.s001.zip › Data/地级市Z_F工作报告.zip!/╣≤╓▌/╫±╥σ╩╨/└┤╫╘╣1⁄2╓┌║┼í╛┬φ┐╦╩2╛▌═°í┐_2013.docx]

各位代表：

现在，我代表市人民政府向大会作工作报告，请予审议，并请列席人员提出意见。

2012年工作回顾

过去的一年，市人民政府在省委、省政府和市委正确领导下，在市人大、市政协的监督支持下，坚持以邓小平理论、“三个代表”重要思想、科学发展观为指导，深入贯彻落实党的十八大和国发〔2012〕2号文件精神，按照省市党代会作出的部署，奋力拼搏、攻坚克难，圆满完成了市四届人大一次会议确定目标，实现了新一届政府工作良好开局。

2012年，预计完成地区生产总值1343.9亿元，同比增长（下同）15.9%；三次产业结构为13.5：45.1：41.4；全社会固定资产投资1306.5亿元，增长60.6%；规模工业增加值554.7亿元，增长22.1%；社会消费品零售总额409.9亿元，增长16.9%；财政总收入288.3亿元，增长42.6%；公共财政预算收入113亿元，增长33.5%；城镇居民人均可支配收入19748元，增长13.3%；农民人均纯收入6061元，增长16.2%。金融机构存、贷款余额分别达1712.2亿元、927.6亿元，比年初增长22.1%、30.3%。“四大区域”竞相发展，县域经济实现增比进位，我市获全省综合测评第1位。

——强力推进“三化同步”战略，发展质量和效益明显提升。新型工业化步伐加快。建成园区标准厂房60万平方米、在建64.3万平方米，新投产项目103个。7个园区列入全省“511示范园区”。长征风电、盘江海螺水泥、裕能科尔微电子等新建项目和茅台酒厂、天蕴纺织等技改项目建成投产，务川氧化铝、绥阳煤电化、九鼎车辆等项目开工，桐梓电厂、二郎电厂等项目扎实推进。完成工业投资550亿元。降低煤调基金征收标准和停征二次煤调基金，争取和发放原材料企业电价补贴近2亿元。城镇化进程加速推进。出台支持遵义国家级经济技术开发区、南部新城加快发展的政策措施。建投大厦、湿地公园、遵义四中及遵义医学院新蒲校区等项目加快推进，东南大道、高新快线、汇川大道延长线等骨干路网加快形成。县城“倍增计划”全面实施。茅台等示范镇建设全面启动。完成城乡建设及房地产投资710亿元。城镇化率达38.9%。农业现代化迈出坚实步伐。粮食、油菜籽总产量分别完成276.2万吨、24.2万吨，种植蔬菜224万亩、辣椒176.1万亩，新增茶园18万亩，收购烟叶202万担。思达、以晴集团在遵项目加快推进，新增市级以上龙头企业50家。实现一产增加值181.2亿元，增长8.9%。湄潭国家农业科技园区挂牌，获“全国十大茶叶生产发展示范县”称号；正安、凤冈茶叶进入欧盟市场；正安“中国白茶之乡”品牌申报成功；桐梓、遵义县获“全国无公害蔬菜生产示范基地县”称号。

——着力建设“三宜”城市，遵义知名度和影响力不断提升。宜居城市建设迈出新步伐。投入5.7亿元实施“双创一巩固”工程。出台公共场所禁止吸烟管理办法、中心城区不文明行为管理试行办法。“国家卫生城市” 通过复检。“全国文明城市”测评排名提升7位。编制中心城区综合交通规划。交通疏解、老城区环境整治、湘江河滨景观整治等取得实效，棚户区改造有序推进。启动100平方公里山体公园建设。凤冈列为“全国生态文明重点宣传区”，湄潭、赤水、正安获省级生态县命名。全市森林覆盖率达48.6%。中心城区空气质量优良率达98.9%。中心城区、县城集中式饮用水源地水质达标率分别为100%、99. 5%。宜业城市建设实现新突破。获 “全国创业先进城市”称号。扶持创办微型企业3900户，带动就业2.1万人。城镇新增就业8.2万人，城镇登记失业率控制在4.2%以内。新增转移农业富余劳动力11.2万人。“国家商标战略实施示范城市”通过复查验收。新增驰名商标4个、地理标志保护产品4个、贵州名牌产品18个。宜游城市建设取得新成效。与省旅游局建立战略合作关系。成功举办全市首届旅游产业发展大会、第四届国际山地户外运动挑战赛。重点推出“红色遵义•醉美丹霞”旅游精品线。新开工旅游项目29个。新增三星级以上酒店6家，评定申报3A级以上景区10个。赤水丹霞地质公园开园。桐梓被授予“国家级休闲农业与乡村旅游示范县”称号。接待游客3354万人次，实现综合收入312亿元。

——全力改善发展条件，基础设施建设持续加强。交通建设实现突破。完成交通投资125亿元。遵义机场建成通航，开通至北京等4条航线；仁怀机场开工。高速公路新增90公里、在建631公里、通车里程达303公里，县县通高速公路项目全部开工。建成通村公路553公里、通村油路1303公里。楠木渡、沿江渡、河闪渡码头投入运行。遵义公路旅游客运中心、中心城区客运枢纽、乡镇客运站加快建设。城市公共客运运力增加，智能化监管系统投入运行。水利建设持续推进。完成水利投资26.5亿元。正安石峰、仁怀石板塘水库下闸蓄水，遵义中桥、桐梓新桥、凤冈河头水库实现截流，遵义灌区（一期）、道真大沙河、绥阳后水河等工程顺利推进，汇川麻沟、余庆魁龙、仁怀大沙坝等骨干水源工程开工建设。解决农村57万人饮水安全问题。实施54座病险水库除险加固，治理中小河流22条、水土流失面积240平方公里，完成10个小型农田水利重点县年度建设任务，新增、恢复、改善灌溉面积35万亩。遵义县绿竹坝、凤冈响滩子等重点烟水工程开工建设。完成烟水配套工程10万亩。水利水电工程移民工作有序推进。市场基础设施不断完善。黔北汽车国际博览城（一期）、富邦家装商城、星力百货城正式营业。遵义国际商贸城、中国茶城、绥阳农产品综合商贸城、凤冈有机食品城等项目有序推进。改造中心城区农贸市场32个。“新网工程”实现行政村全覆盖。新建粮食仓容31.5万吨。电力、信息基础设施建设加强。完成电力投资22.4亿元，500千伏诗乡输变电等工程加快实施。“天网工程”顺利推进；完成63个空白乡镇邮政局（所）补建工作；乡乡通宽带、行政村通电话目标基本实现。

——大力推进改革创新，开放开发战略深入实施。重点改革稳步推进。农村产权制度、中小企业融资、事业单位、矿产资源配置等改革取得突破。启动6个县级公立医院综合改革试点。“国家公共文化服务体系示范区”创建取得成效，文化体制改革获文化部表彰。邮政体制改革深入实施，遵义市邮政管理局挂牌成立。开放合作全面推进。与重庆綦江、北京海淀结为友好城市。遵渝合作取得积极进展，桐梓重庆工业园建设取得实效。与北大信息科技学院、浙江大学、西南大学、河海大学等签订市校合作协议。与农行、农发行、交行、农信社省级机构签订银政合作协议，浦发、招商、重庆等银行分（支）行挂牌营业，贵州茅台集团财务公司成立，中信银行遵义分行加快筹建，市投资集团公司等发行企业债券48亿元。与茅台、保利、中电投、中建等企业（集团）合作更加紧密。遵义国家级经济技术开发区正式挂牌，省级经济开发区达到11个。引进省外到位资金712亿元，增长40%；完成进出口总额3.1亿美元，增长21.7%；实际利用外资1亿美元，增长203%。

——倾力办好民生实事，和谐遵义建设迈出新步伐。社会事业全面发展。实施各类教育工程1696项，其中新（改、扩）建乡镇公办幼儿园92所；40余万名农村义务教育学生受益营养午餐改善计划；与32所省外高中协作发展，高中阶段毛入学率达62.3%；遵义医专通过省级示范院校评审。乡村卫生服务一体化全面推行；村医待遇补助政策全面落实；食品药品安全、医疗卫生监督、重大疾病防控、爱国卫生运动、妇幼保健工作全面加强。连续6次荣获“全国无偿献血先进城市”称号。人口出生率、自增率分别控制在10.5‰、5.1‰以内。获省级以上科技及知识产权立项276项；专利申请量、授权量分别突破2000件、1000件；省级娄山关高新技术产业开发区申报成功；国家级茶及茶制品、低压电器检测中心获筹建批文。“15851”人才工程深入推进。大型舞台剧《传奇遵义》赴京演出获好评；海龙囤列入中国世界文化遗产预备名单；遵义作家获全国少数民族文学创作“骏马奖”。《遵义百科全书》出版发行。遵义籍运动员卫冕奥运会冠军；成功举办全省第二届农运会，遵义代表团获金牌、奖牌数第一。改造农村危房4.3万户；新开工保障性住房1.8万套、竣工1.7万套；1.8万户低收入家庭享受廉租住房保障；新农保和城镇居民社会养老保险实现全覆盖。城市低保标准人均月增33元，农村低保标准人均年增186元。完成生态移民搬迁4320户/1.84万人。习水县及全市20个重点乡镇“减贫摘帽”，20 万人脱贫。“十八件民生实事”完成投资55亿元。社会管理创新持续加强。安全生产事故起数、死亡人数分别下降29.1%、36.2%，连续12年实现“双降”。政法基础设施、城市报警监控及智能交通指挥平台等项目全面启动；社区戒毒“阳光工程”全面实施。遵义“四在农家•美丽乡村”建设模式、基层矛盾化解“余庆经验”在全省推广；信访维稳、应急处置各项工作得到加强；社会治安打防控体系逐步完善，群众安全感达92.4%。渝鄂湘黔桂民族工作边区联席会议在赤水成功召开。连续七次荣获“全国双拥模范城”称号。外事侨务、人防战备、国防教育、水文、气象、地方志、档案、老龄人、残疾人、红十字等事业全面发展。

——努力加强政府自身建设，政府职能加快转变。修订完善《政府工作规则》，出台促进效率提升的意见，完成国发〔2012〕2号文件课题研究20个。办理人大代表建议165件、政协委员提案428件，办结率达100%，满意和基本满意率为99.5%。行政复议试点工作加强，行政处罚自由裁量权不断规范。网上行政审批和电子监察作用充分发挥。市级行政许可审批事项从151项减少到72项、非行政许可审批事项从13项减少到10项。在全省率先建成市级公共资源交易中心，审计监督、行政监察和行政问责力度加大，廉政建设得到加强。

各位代表，过去一年，在国际国内经济形势错综复杂的情况下，能够取得这些成绩，得益于省委、省政府和市委的坚强领导，得益于市人大、市政协的监督支持，得益于全市广大干部群众的艰苦奋斗。特别是通过承办全省重点项目建设观摩会、小城镇建设推进大会、茶产业发展大会、创业型城市建设现场会等，全面展示了遵义形象，提振了发展信心。在此，我代表市人民政府，向全市人民、广大干部职工，向各民主党派、工商联、无党派人士、人民团体，向驻遵解放军指战员、民兵预备役人员、武警消防官兵、公安干警，向历届老领导老同志，以及所有关心支持遵义经济社会发展的各界人士，表示衷心的感谢！

各位代表，过去一年的经验值得认真总结。实践表明：遵义要实现“两个率先”，必须更加解放思想，改革开放，创新发展；必须更加瞄准一流，增比进位，跨越发展；必须更加突出重点，统筹兼顾，全面发展；必须更加务实高效，强化执行，保障发展。

在充分肯定成绩的同时，我们也清醒地看到，遵义的发展还存在不少突出矛盾和问题，主要体现在：经济总量不大，人均水平不高，城乡居民收入整体水平仍然较低；制约发展的瓶颈仍然存在，推进项目建设的用地难、融资难等问题仍然突出；财政整体较弱，支出压力较大，集聚财力办实事、抓发展实力仍不强；干部作风有待加强，执行落实还有差距，发展环境还不够宽松等。我们将在今后的工作中，采取更加有力的措施，用发展的办法予以解决。

2013年主要工作任务

今年是“同步小康创建活动”启动之年，也是实施“十二五”规划的关键之年。总体看，宏观经济形势趋好，发展面临重大新机遇，但困难和问题不容忽视。我们要坚定战略部署不动摇、坚定目标任务不懈怠、坚定思路政策不折腾，增强发展自信、跨越自信、小康自信，团结拼搏，真抓实干，把同步小康宏伟事业不断推向前进。今年政府工作的总体要求是：坚持以党的十八大精神为指引，认真落实中央、全省经济工作会和省委十一届二次、市委四届二次全会精神，紧紧围绕实施“三大战略”、建设宜居宜业宜游美丽遵义，以“全力追赶、力争超越，努力实现两个率先”为总基调、总目标，以“稳中求快、快中争先，快中求好、更好更快”为总要求，以提高经济增长质量和效益为中心，以转变经济发展方式为主线，进一步解放思想、改革开放、凝聚力量、攻坚克难，全力推动经济社会发展实现新突破。

今年全市经济社会发展的主要预期目标是：完成地区生产总值1620亿元，增长17%。其中：一产增长7%，二产增长21%，三产增长15%。规模工业增加值700亿元，增长23%以上。全社会固定资产投资2000亿元，增长53.3%。社会消费品零售总额492亿元，增长20%。财政总收入360亿元，增长25%；公共财政预算收入141亿元，增长25%。城镇居民人均可支配收入22908元，增长16%。农民人均纯收入7091元，增长17%。城镇登记失业率控制在4.2%以内。城镇化率达41%。单位生产总值能耗和地耗、主要污染物排放总量控制在省下达指标内。

围绕上述目标，我们将重点抓好以下工作：

一、统筹推进四大区域发展，着力在全面建成小康社会征程中迈出新步伐

深入开展全面小康创建活动。按照“党委领导、政府负责，市域统筹、县为单位，社会协同、全民参与”的要求，坚持“三个不能代替”，突出“三个核心指标”，强化预警监测，健全完善以地理区位、经济水平为基础，与全面小康指标体系相匹配的目标考核体系。结合乌蒙山、武陵山片区区域发展与扶贫攻坚规划，加大对发展相对滞后地区的政策、项目扶持。完善年度工作推进计划目标，以阶段工作目标确保整体目标实现，全面小康创建速度高于遵义以往、高于全省、高于西部，为在全省走前列、在西部同类城市位次前移和实现“两个率先”奠定坚实基础。

分类指导梯次推进全面小康。统筹“四大区域”发展。推进中部区域加速崛起，着力打造黔北经济协作区加快发展的“火车头”、“三宜”城市示范区和全面小康示范县。推进西部区域快速突破，努力将赤水河流域打造成为“两个率先”的发动机、世界著名的旅游精品线和遵义发展新的增长极。推进东部区域提速开发，着力建设绿水青山、田园风光式的新农村示范区和生态文明示范县。推进北部区域全速攻坚，力争发展速度赶上甚至超过全市平均水平，着力创建扶贫开发攻坚示范区。继续推进强县强镇建设，鼓励发展基础好的县率先实现小康，支持发展中等的县尽快实现小康，帮助发展困难的县如期实现小康。

二、加快推进新型工业化，着力在产业园区发展上实现新突破

强力推进园区建设。围绕全省重点打造“100个产业园区”总体布局，深入推进19个重点园区建设，完成园区总体布局和专业性规划。按照产城同步发展思路，实施园区核心区“七通一平”基础设施建设攻坚，实现城镇基础设施、公共服务体系向园区延伸和配套，提升园区集聚能力和承载能力。探索园区持续投入和偿债机制，建立园区梯次提升机制。完善园区管理体制，以任命与聘用等相结合充实队伍，采取“以奖代补”等激励措施增强创建活力。完成园区投资 380 亿元以上，开工、投产项目各100个以上，建成标准厂房70万平方米以上。

强力推进产业发展。着力抓好“十大工业产业”发展，做大做强特色优势产业，推进特色轻工业集群化品牌化、装备制造业整机化高端化、材料产业一体化高新化、能源及煤电化一体化精细化发展，加速产业链延伸，提高附加值。加大名优白酒企业技改力度，稳步扩大产能；整治仁怀白酒小作坊，优化重组白酒资源；加快建设赤水河白酒产业长廊，大力培育“中国酱香•赤水河谷”地域品牌，实现白酒产量40万千升以上。优化“两烟”结构，实现卷烟生产55万大箱以上。加快煤、铝等资源勘探开发步伐。积极实施煤矿整合重组，实现煤炭产量1200万吨以上。强化工业运行调度，继续采取电价补贴、丰枯电价扶持等措施，保障工业企业稳定发展，扩大规模工业在全省的领先优势。

强力推进项目建设。建立重大项目风险评估、防范机制，严把环评关，实现经济与环境协调发展。落实项目建设“五个一”工作机制，强化跟踪管理，加强协调服务。加快煤及煤化工、铝及铝加工、钛及钛加工等项目建设。力争热电联产动力车间项目建设取得突破。建成投产贵州钢绳异地技改项目（一期）、航天汽车、九鼎车辆、桐梓电厂等项目，加速推进务川煤电铝、绥阳煤电化、正道煤电铝、习水二郎电厂等项目，开工建设遵义卷烟厂、汇兴铁合金等异地技改项目。完成工业（含园区）投资700亿元。

三、加速推进绿色城镇化，着力在扩容提质上实现新突破

提高综合承载能力。坚持以工业园区和城镇新区为载体，推进产城融合，把中心城区和县城作为迅速推进城镇化、加速全面小康的突破口，推动城镇化和工业化良性互动。加快遵义县、绥阳县撤县设区、同城化发展和桐梓县、湄潭县撤县设市步伐，大力建设鸭溪等卫星城市，把中心城区建成200万人口区域性中心城市，预留300万人口以上城市发展空间。加快打通连接新城区与老城区的城市主干道、新老城区与高速公路的快捷通道，着力推进城市主干道沿线开发建设，形成连接新老城区的商贸廊道。围绕全省打造“100个城市综合体”要求，深入推进18个城市综合体项目。强力推进新蒲新区建设，建成新蒲新城路网、新火车站片区路网，完成建投大厦等“十大标志工程”。加速推进南部20平方公里新城建设，完善路网、电网、管网等基础设施，配套建设高星级酒店，积极引进教育和医疗机构，大力发展商贸业和总部经济。加快推进北部32平方公里新城建设，基本形成高坪片区路网。着力完善中心城区市政设施，加快100平方公里森林公园建设，继续实施老城、东联线等交通疏解工程，推进18条骨干道路建设。加快老工业区、棚户区、城中村改造步伐。深入实施县城“倍增计划”，加快学校、医院等设施建设，完善县城功能。启动汇川至桐梓、汇川至绥阳、绥阳至新蒲、新蒲至湄潭城市干道或快速通道建设，推动同城化发展。完成城乡建设及房地产投资900亿元。

精心打造示范小城镇。设立小城镇建设专项基金，强化部门挂帮责任，重点规划建设54个布局合理、设施配套、交通方便、功能齐全、环境优美的“六型”特色小城镇，努力在全省“100个示范镇”建设中走前列、作表率。完成小城镇建设投资230亿元以上。按照“贵州第一、全国一流、世界知名”的定位，加快将茅台镇打造成为中国国酒之心、中国历史文化名镇、世界旅游名镇。

提升城镇管理水平。加快数字遵义建设，大力推进城镇各类信息资源共享，实现城市管理信息化、精细化。以城郊结合部为重点，加大统一规划建设力度，依法打击违法建设，维护规划严肃性、强制性。加强交通整治，合理设置交通设施，科学布局停车场建设，提升公交车、出租车运营质量和服务水平。继续巩固“国家卫生城市”、“国家园林城市”创建成果。合理布局城市绿色空间。实施中心城区车行立交桥、人行天桥、农贸市场及湘江河（二期）、洛江河、忠庄河、高坪河等景观改造工程。加大重点区域治理力度，为2014年成功创建“全国文明城市”夯实基础。

四、扎实推进农业现代化，着力在调结构促增收上实现新突破

夯实农业发展基础。落实强农富农惠农政策，毫不放松粮食生产，稳定耕地面积。加快土地整理整治和中低产田土改造，实施千亿斤粮食增产工程。提升山区农业机械化水平。拓宽农业保险领域。加强人工影响天气及气象防灾减灾体系建设。加快市农科所所改院和整体搬迁。新增50家市级以上龙头企业，打造18个省级现代高效农业示范园区和100个市级农民专业合作示范社。新建农民专业合作社50个、村级综合服务站120个，改造重组基层供销社13个，组建行业协会18个。规模以上农产品加工业总产值达200亿元。

壮大特色优势产业。大力实施“853”工程及农民收入过万元工程。新增茶园面积20万亩，种植酒用高粱120万亩、辣椒175万亩。完成粮食总产量300万吨左右、肉类总产量55万吨，收购烟叶200万担。狠抓蔬菜基地建设，构建蔬菜直销网络，保障蔬菜供给。加大“四种工程”建设力度，提升农业新品种新技术繁育试验示范水平。大力发展无公害、绿色、有机和地理标志保护产品，建设农产品质量安全监测网络，提高农产品质量安全水平。

构建繁荣和谐新农村。进一步完善被征地农民就业和社会保障制度。大力开发农村劳动力资源，结合园区企业需求，加强农民技能培训，新增转移劳动力10万人。落实好生态移民、大中型水利水电工程移民及后期扶持政策。支持农民工返乡创业，鼓励发展林下经济、中药材种植、乡村旅馆、家庭农场等。充分发挥“四在农家•美丽乡村”品牌效应，着力向纵深推进，新增创建15万户、70万人受益。

五、大力发展第三产业，着力在做大总量提升质量上实现新突破

加快发展信息产业。围绕建设西部重要的电子元器件研发生产基地、西南地区新型电子材料和锂离子电池产业集聚区、呼叫中心基地、软件认证基地的目标，加速构建以电子信息制造、软件开发、新一代通信和信息技术服务为主的产业体系，培育新的增长极。继续实施电子政务提升工程、“三农”信息服务工程，强化信息网络安全，扎实推进信息3G、无线城市等建设。加大“智慧遵义”建设力度，启动市政府云计算中心、社会公共服务平台建设。入驻信息产业企业20户以上、投产10户以上。完成信息产业投资10亿元以上。

全力推进文化旅游产业大发展。编制全市文化发展与保护总体规划，推动文化、旅游衔接发展，夯实宜游城市建设基础。加快茅台、土城、苟坝、沙滩、赤水文化旅游创新区和长征文化博览园、赤水景区、中国茶海景区建设，支持各地竞相发展文化旅游创新区。深度开发娄山关景区。打造《传奇遵义》常态演出精品剧目和中国长征、茅台酒、仡佬、茶等文化旅游节。深入挖掘“知青”、“三线”建设等文化旅游资源。做大做强文化旅游产业发展投融资平台。加快推进遵义会议陈列馆改扩建工程、飞龙湖国家湿地公园、市级综合旅客接待服务中心、县级数字影院建设。继续推进遵义报业、广电传媒等文化企业集团化发展。提档升级办好第二届遵义旅游产业发展大会。加快星级酒店建设。大力发展乡村旅游。集中打造“红色遵义、人文遵义、醉美遵义”旅游形象品牌、“七大旅游品牌”、17个精品景区。确保旅游接待人次、综合收入分别增长25%和30%以上。

积极推进相关产业升级发展。出台措施加快第三产业发展。以城市综合体建设为契机，加快中央商务区建设，促进房地产业健康发展。加大“引金入遵”力度，引进2家全国股份制银行入驻，加快组建农村商业银行，大力发展小额贷款、融资性担保公司和村镇银行。推动遵义国际商贸城、黔北国际汽车博览城（二期）、新雪域冷链物流、中国茶城等项目建设。建成“万村千乡”市场513个、农村配送中心17个，推进县乡农贸市场和“农超对接”建设。支持会展、电子商务、中介咨询、科技研发、创意、教育培训等服务业加快发展。

六、加速完善基础设施，着力在改善发展条件上实现新突破

提速交通基础建设。加密遵义机场航线，加快仁怀机场建设和黔北机场前期工作。实现杭瑞高速思遵段遵义境、仁怀至赤水高速、绕城高速东北段建成通车，全市高速公路通车里程突破600公里。加快推进贵阳至遵义复线、遵义北环檬乐段和道真至瓮安、余庆至凯里、务川至正安等高速公路和中心城区连接遵义机场城市主干道建设。做好正安至习水、余庆至花山、习水至古蔺、绥阳至凤冈高速公路项目前期工作。加快推进沿边公路建设，打通融入川渝通道。协助推进渝黔快铁、昭黔铁路建设，做好川黔铁路李家湾至阁老坝段东移前期工作。完成构皮滩库区大坝翻坝运输、乌江三级航道扩能、赤水河上游航道延伸等工程，加快乌江渡、石垭子、桐梓河、芙蓉江流域等库区及湄江河水运工程前期工作。推进城市公共交通优先发展，建设公交都市。完成遵义旅游客运中心、中心城区公共客运监管系统建设。启动城乡客运一体化、中心城区轨道交通规划。加快农村公路改造提等升级。完成交通建设投资150亿元。

突出水利建设重点。完成遵义灌区（一期）、道真大沙河水库项目；加快遵义中桥、习水铜灌口等工程建设；开工建设遵义县上水、赤水金龙等骨干水源及烟水工程；启动湄凤余灌区（四期）、遵义灌区续建配套工程、苟浒引水工程；完成63座病险水库除险加固；加快“五小”水利建设；治理中小河流26条和水土流失面积240平方公里；完成11个小型农田水利重点县年度建设任务，新增、恢复改善灌溉面积35万亩；新建水文站27个。完成水利建设投资35亿元。组建市水务投资有限公司。进一步推进中心城区、仁怀市、遵义县水务一体化改革试点工作。

加快能源电力设施建设。继续实施电网改造，完成电力投资15.1亿元以上。完善以500千伏变电站为支撑、220千伏电网为骨架的城乡输配电网络。健全产业园区、城市新区电网设施。加快推进凤冈、湄潭、绥阳页岩气勘探开发。启动道真角木塘和习水兰子口电站建设。开展风力发电基站测试，开工建设正安、桐梓风电项目。加快遵义县液化天然气站建设。建成农村沼气池5500口及服务网点70个、清洁工程示范点20个。

七、坚定不移推进改革开放，着力在激活发展动力上实现新突破

深化重点领域改革。深入开展“改革开放年”活动，为遵义加快发展提供不竭动力。深入实施农村综合配套改革，扩大湄潭农村土地制度改革和凤冈“三资转换”试验成果，加大集体土地、集体林权、宅基地和房屋产权、其它动产等产权改革力度，破解农村发展难题。做大做强融资平台，优化金融生态环境，完善银企沟通协调机制，着力破解中小企业融资难题。深化新型农村合作医疗、国家基本药物制度、医疗卫生服务体系、公共卫生服务项目重点领域改革，全面推进县级公立医院综合改革试点。按照政事分开、事企分开和管办分离的要求，扎实推进事业单位分类改革试点。充分发挥市级公共资源交易平台作用，大力推进招投标管理改革。坚持政府调控资源、市场配置资源原则，探索建立地质找矿新机制，切实推进矿产资源市场清理和资源整合。深入实施财税体制改革。全面开展“综合治税”工作。加强预算管理，实现开源节流。加快推进统筹城乡综合配套改革试点。继续推动户籍制度、国有企业、科技文化、教育体制等重点领域改革。

大力发展开放型经济。加大招商引资力度，强化项目储备，创新招商方式，优化投资环境，保护投资人合法权益，确保引进省外资金926亿元以上、增长30%以上。深化遵渝战略合作，加强与上海对口帮扶合作。落实经济开发区发展扶持措施，培育百亿元级、千亿元级开发区，实现县县有省级开发区目标。申报具有保税功能的特殊监管区、国家外贸转型升级示范基地、遵义航空口岸和娄山关国家级高新技术产业开发区，支持红花岗、仁怀经济开发区申报国家级经济技术开发区。外贸进出口总额增长25%，实际利用外资总额增长30%。

发展壮大民营经济。继续完善市级领导挂帮重点民企和联席会议制度。扶持行业协会发展，着力发挥其自主管理功能。落实中小微企业发展政策，加大扶持力度。大力实施“中小企业成长”和“万户小老板创业”工程，建成一批具有示范效应的省级民营企业。不断提高民营经济在国民经济中的比重。

八、加强生态环境保护，着力在美丽遵义建设上实现新突破

推进创模工作。切实加强赤水河、乌江等重点流域污染防治和生态建设。加强自然保护区、国家森林公园、湿地公园建设管理。实施中心城区饮用水源环境综合整治，推进各地饮用水源一级保护区硬隔离设施建设。加大重点企业环境监察执法力度，强化风险源管理和废水、废气等污染防治。着力解决损害群众健康的突出环境问题，保障环境安全。完成人工造林55万亩、封山育林20万亩。在中心城区建成5个PM2.5监测点，建立监测发布体系。确保县级以上集中式饮用水源地水质达标率达100%、中心城区环境空气质量优良率达90%以上。力争国家环保模范城市通过省级验收。努力建设天蓝、地绿、水净的美好家园。

促进节能减排。启动国家新能源示范城市创建工作。加大落后产能淘汰力度。落实节能减排责任制，加强企业用能计划管理，推广节能新技术及新产品。鼓励重点企业和产业园区发展循环经济。推进垃圾及污泥利用水泥窑协同处置项目建设。建成东部污水处理厂。加强污水处理设施运行管理。加强农村面源污染治理。探索建立生态补偿机制。加大规模畜禽养殖场（小区）污染治理力度。加快县（市）机动车尾气检测站建设，确保检测率达80%以上。

加强耕地保护。完成土地整治规划编制，开展耕作层保护利用、土地流转及增减挂钩试点等工作，加强低丘缓坡等未利用土地开发利用，发展“工业梯田”，探索山地城镇建设路子，加大土地投入强度。

九、坚持不懈发展民生事业，着力在创新社会管理上实现新突破

加强和创新社会管理。扎实开展“六五”普法。总结推广 基层矛盾化解“余庆经验”。实施关爱外出民工、关爱留守儿童、关爱空巢老人“三关爱工程”。继续推进“平安遵义”创建，防范和打击违法犯罪活动。加强刑释解教人员、违法青少年等特殊人员帮教管理。深入推进社区戒毒康复“阳光工程”。建成城市报警与监控“天网工程”暨智能交通管理指挥平台、市强制戒毒劳教所项目。强化社区服务功能，深入推进“四民社区”、“农村社区建设全覆盖”创建工作。夯实安全生产基层基础工作，严格执行安全生产“党政同责”、“一岗双责”制度，落实生产经营单位主体责任，推进安全生产领域“打非治违”专项行动，强化企业质量安全标准化建设。继续实现安全生产事故起数和死亡人数“双降”目标。加强重点领域价格监管，强化食品药品安全质量管理，有效维护市场经济秩序。

大力发展社会事业。优先发展教育，规范教育教学管理，推进义务教育、学前教育、民办教育、特殊教育、职业教育协调发展，启动遵义大学筹建，支持茅台学院建设。完善市县乡三级医疗卫生服务体系，增加每千人口拥有卫技人员数和开放床位数，提高基层医疗卫生队伍服务能力。加强食品药品、疾病预防、卫生监督、妇幼保健、卫生应急处置、血液安全保障等能力建设，实现城镇居民医保市级统筹，提高报销比例。探索建立失独家庭社会保障制度。扎实推进流动人口综合服务管理和出生人口性别比综合治理，人口出生率、自增率分别控制在10.4‰、5.1‰以内。深入实施 “15851”人才工程，进一步完善人才引进、培养、任用机制。启动汇川、正安、余庆、习水、绥阳、仁怀就业保障综合服务平台建设，综合利用“3个15万元”、“小额贷款”等政策，扶持高校毕业生、妇女、下岗职工、残疾人等重点人群创业就业。提高全民科学素质。积极参与科技交流与合作，省级以上项目立项240个以上，力争高新技术企业突破50家，申报专利2600件以上。“国家公共文化服务体系示范区”创建通过验收。抓好文化遗产保护与利用。推进海龙囤、“四渡赤水”申遗和青杠坡申报国家级烈士陵园。推进民族团结进步创建活动，促进民族地区加快发展。充分发挥宗教界人士和信教群众的积极作用，深入开展“和谐寺观教堂”创建活动。开展第八次“全国双拥模范城”创建活动。完善统计制度。抓好第三次全国经济普查。促进外事侨务、国防教育，人防战备、殡葬改革、老龄人、残疾人、红十字等事业全面发展。

着力办好民生实事。坚持民生财政、民生预算，优化财政支出结构，增加民生项目比重，加强基本公共服务体系建设，大力发展社会事业，重点办好“十件民生实事”。⑴ 搬迁生态移民1.8万人；实现正安县和20个重点乡镇“减贫摘帽”目标，减少贫困人口20万人以上。⑵改造农村危房4万户，购、建廉租住房3079套，开工建设公共租赁住房1.9万套，实施棚户区改造1.57万户；完成村庄整治2万户。⑶启动 “9+3”义务教育及三年免费中等职业教育计划；继续实施高中阶段教育项目10个；实施农村寄宿制学校学生宿舍项目120个、教师公租房项目150个；新（改、扩）建乡镇公办幼儿园50所。⑷城市低保标准人均月增35元，农村低保标准人均年增184元；建设市社会福利院老年养护楼、流浪未成年人救助保护中心、救助管理站、反家庭暴力避难所；加快基层救助体系建设；建成50所乡镇敬老院，力争新（改、扩）建实现全覆盖。⑸改扩建市第四人民医院，启动市第一人民医院内科大楼、市精神病专科医院和2个县级中医院建设，县级综合医院规范化建设全部达标；完成79所乡镇卫生院标准化建设；新农合政策范围内统筹基金最高支付限额由10万元提高到12万元；全面实施农村儿童先天性心脏病等22种大病保障工作。⑹新建乡镇计生服务站10个、村计生服务室30个，实现免费孕前优生健康检查全覆盖。⑺新增城镇就业7.7万人，安置公益性岗位1.3万个以上，失业人员再就业1.44万人以上，就业困难人员再就业8300人以上；新增微型企业2800户，带动就业1.5万人。⑻实施“一事一议”财政奖补项目893个；建成通村油路1000公里；开工建设颜村综合客运枢纽及2个县级客运站、5个乡镇客运站。⑼解决80万农村人口饮水安全问题。⑽开工建设市劳动人民文化宫、市档案方志馆，建成汇川、红花岗、仁怀、道真、务川及遵义县档案馆；新建30个乡村学校少年宫、25个农民文化家园；新增10.4万座农村广播电视直播卫星地面接收站；新建45个村级、6个乡镇农民体育健身工程和40个全民健身“路径工程”。

十、加强政府自身建设，努力在建设服务型政府上实现新突破

增强创新意识，推动思想解放。坚持“十破十立”，深入开展解放思想大讨论活动，使解放思想更实际、更实在、更富实效。通过思想解放，激发创新活力，增添发展动力，敢想敢干、敢作敢为、敢比敢攀、敢争敢赛，在全市上下营造增比进位突破、创业创新创优的浓厚氛围，形成集中精力谋发展、抓发展的强大合力。

增强服务意识，推动作风转变。认真落实关于作风建设的各项规定，深入开展“效率建设年”活动，纠正部门和行业不正之风，着力解决工作不落实、不兑现、不作为和文件多、会议多、应酬多、不在状态等问题，狠刹“庸、懒、散、奢”等不正之风，促进工作在一线落实、问题在一线解决，确保各项工作落到实处。

增强法治意识，推动环境改善。坚持依法行政，着力提高各级干部运用法治思维和法治方式深化改革、推动发展、化解矛盾、维护稳定的能力，努力营造办事依法、遇事找法、解决问题用法、化解矛盾靠法的公平正义的法治环境。加大政务公开力度。自觉接受市人大的法律和工作监督、市政协的民主监督及新闻媒体、社会公众的舆论监督。

增强廉政意识，推动发展提速。落实廉政建设责任制，推进制度建设，着力从源头上防治腐败。加强经费管理，树立节俭意识，控制和减少“三公”经费支出，集中财力办大事。加强行政监察和审计监督。严格追究失职渎职行为，严肃查处各种违纪违法案件，努力营造干部清正、政府清廉的工作环境。

各位代表，在开启全面建成小康社会的新征程中，我们面临的任务艰巨而繁重，肩负的使命神圣而光荣。让我们紧密团结在以习近平同志为总书记的党中央周围，在省委、省政府和市委的坚强领导下，团结和依靠全市各族人民，励精图治，攻坚克难，全力推动经济社会又好又快、更好更快发展，为在全省率先全面建成小康社会而不懈奋斗！
